# Effect of household air pollution due to solid fuel combustion on childhood respiratory diseases in a semi urban population in Sri Lanka

**DOI:** 10.1186/s12887-019-1674-5

**Published:** 2019-09-02

**Authors:** Nayomi Ranathunga, Priyantha Perera, Sumal Nandasena, Nalini Sathiakumar, Anuradhani Kasturiratne, Rajitha Wickremasinghe

**Affiliations:** 1 Faculty of Medicine, University of Kelaniya, P.O. Box 6, Thalagolla Road, Ragama, 11010 Sri Lanka; 2 National Institute of Health Sciences, Kalutara, Sri Lanka; 3 Department of Epidemiology, School of Public Health, University of Alabama at Birmingham, Birmingham, USA; 4 Department of Public Health, Faculty of Medicine, University of Kelaniya, Ragama, 11010 Sri Lanka

**Keywords:** Household air pollution, Respiratory infections, Children under 5, Biomass fuel, Sri Lanka

## Abstract

**Background:**

Household air pollution from combustion of solid fuels for cooking and space heating is one of the most important risk factors of the global burden of disease. This study was aimed to determine the association between household air pollution due to combustion of biomass fuel in Sri Lankan households and self-reported respiratory symptoms in children under 5 years.

**Methods:**

A prospective study was conducted in the Ragama Medical Officer of Health area in Sri Lanka. Children under 5 years were followed up for 12 months. Data on respiratory symptoms were extracted from a symptom diary. Socioeconomic data and the main fuel type used for cooking were recorded. Air quality measurements were taken during the preparation of the lunch meal over a 2-h period in a subsample of households.

**Results:**

Two hundred and sixty two children were followed up. The incidence of infection induced asthma (RR = 1.77, 95%CI;1.098–2.949) was significantly higher among children resident in households using biomass fuel and kerosene (considered as the high exposure group) as compared to children resident in households using Liquefied Petroleum Gas (LPG) or electricity for cooking (considered as the low exposure group), after adjusting for confounders. Maternal education was significantly associated with the incidence of infection induced asthma after controlling for other factors including exposure status. The incidence of asthma among male children was significantly higher than in female children (RR = 1.17; 95% CI 1.01–1.37). Having an industry causing air pollution near the home and cooking inside the living area were significant risk factors of rhinitis (RR = 1.39 and 2.67, respectively) while spending less time on cooking was a protective factor (RR = 0.81). Houses which used biomass fuel had significantly higher concentrations of carbon monoxide (CO) (mean 2.77 ppm vs 1.44 ppm) and particulate matter_2.5_ (PM_2.5_) (mean 1.09 mg/m^3^ vs 0.30 mg/m^3^) as compared to houses using LPG or electricity for cooking.

**Conclusion:**

The CO and PM_2.5_ concentrations were significantly higher in households using biomass fuel for cooking. There was a 1.6 times higher risk of infection induced asthma (IIA) among children of the high exposure group as compared to children of the low exposure group, after controlling for other factors. Maternal education was significantly associated with the incidence of IIA after controlling for exposure status and other variables.

## Background

Household air pollution from combustion of solid fuels for cooking and space heating is one of the ten most important risk factors of the global burden of disease [1]. Household air pollution contains some of the same pollutants found in tobacco smoke and in ambient air which have been linked with serious health consequences. There is compelling evidence linking household air pollution to acute respiratory infections in children [2]. There is growing evidence that high household air pollution caused by cooking with biomass is a major hazard that seriously affects children and the elderly [3].

In 1981 and 2012, firewood was the principal type of cooking fuel used in 94 and 78% of households in Sri Lanka, respectively [4]. Most of the local stoves used traditionally for firewood have incomplete combustion resulting in high pollutant emissions [5].

Respiratory tract infections and other respiratory tract diseases are responsible for a considerable proportion of morbidity and mortality worldwide [6]. Pneumonia is the one of the leading causes of death in young children and half of deaths due to pneumonia is due to air pollution [7]. Exposure to biomass smoke is strongly associated with acute respiratory tract infections in preschool children worldwide.

The most vulnerable age group for health hazards from household air pollution are children under 5 who live in the house with their mother and are exposed to polluted air due to combustion of unprocessed biomass fuel. They are more affected than adults as they inhale large amounts of polluted air compared to their body size due to increased minute ventilation as they are more active. They breathe more polluted air than adults as they breathe the air closer to the ground where more particulate matter concentrates [8].

A study in Japan revealed that the use of wood for cooking is a risk factor for respiratory infections in children and women who spend more time inside the kitchen when the stove is lit [9]. A systematic review and a meta-analysis have reported that the prevalence of pneumonia in children in households using solid fuel is higher that in children in households not using biomass fuels [2].

A cross-sectional survey done in Brazil reported an acute lower respiratory illness prevalence of 23.9% among 771 children living in houses using solid fuels. The main risk factors were previous episodes of acute lower respiratory tract infection or wheezing, crowding, maternal schooling less than 5 years, monthly family income less than US$ 200, 4 or more people sleeping in a room, asthma in family members, and maternal smoking [10].

A meta-analysis done in 2011 revealed that the prevalence of acute respiratory infections in children exposed to household air pollution due to solid biomass fuel combustion is three times higher than in non-exposed children [11].

The aim of this study was to evaluate the relationship between household air pollution due to solid fuel combustion and self reported childhood respiratory tract diseases among children under 5 in the Ragama Medical Officer of Health (MOH) area in Sri Lanka.

## Methods

### Study design

This prospective study in which children under 5 were followed up for 12 months was conducted in the Ragama Medical Officer of Health (MOH) area in Sri Lanka from June 2011 to April 2014.

### Study setting

The Ragama MOH area is situated in the Gampaha district of Sri Lanka, the second most populous district of the country having an estimated population of 2.3 million with a population density of 1719/km^2^ in 2012 [4]. It has urban and semi-urban to rural characteristics with a multi-ethnic population. According to the census of population and housing conducted in 2012, approximately 63% of households in the Ragama MOH area used biomass fuel and 31% used LP gas [4].

### Study population and sampling method

The study population comprised children under 5 who were permanent residents of the Ragama MOH area. This study was an extension of a larger study investigating the effects of exposure to solid fuel smoke during pregnancy on birth outcomes. Six hundred and fifty pregnant females from the Ragama MOH area were recruited for the parent study.

The sample size was calculated based on the following formula [12]:

$$ \mathrm{n}={\left\{\ {\mathrm{Z}}_{1-\upalpha /2}\surd \left[2\mathrm{P}\left(1-\mathrm{P}\right)\right]+{\mathrm{Z}}_{1-\upbeta}\surd \left[{\mathrm{P}}_1\ \left(1-{\mathrm{P}}_1\right)+{\mathrm{P}}_2\ \left(1-{\mathrm{P}}_2\right)\ \right]\ \right\}}^2/{\left({\mathrm{P}}_1-{\mathrm{P}}_2\right)}^2 $$

where

n = Sample size

Z^2^_1- α/2_ – percentile of the standard normal distribution corresponding to a particular alpha error

Z_1-β -_ - percentile of the standard normal distribution corresponding to a particular β error

P_1_ - Probability of disease in children with high exposure (exposed to air pollution due to use of biomass fuel and kerosene)

P_2_ - Probability of disease in children with low exposure (exposed to air pollution due to use of LPG and electricity)

P_1_ - P_2_ Difference between the population proportions

P – average probability of disease.

Based on studies conducted in India [13], Brazil [10] and WHO estimates [14], we assumed that 40% of children in the high exposure group will experience 4 infections in a year and 20% of children in the low exposure group will experience 4 infections a year giving a risk ratio of 2.0. Assuming that the power of the study is 90% and the alpha error is 5%, 109 children in each group (total of 218 children) had to be studied.

From the initial baseline survey, households having children under 5 were identified. There were 262 children under 5 in households in which pregnant mothers were recruited for the larger study. In order to account for potential loss to follow up all children were invited to participate in the study.

All children living in a selected household who were under 5 years of age and whose parents gave consent to participate in the study were included in the study. Children with any congenital abnormality or syndromic disease, with documented immunodeficiency or diagnosed to have any chronic disease other than respiratory diseases were excluded.

### Data collection

All eligible households with a child under 5 were identified at the time of recruitment of pregnant females into the larger study on the effects of exposure to solid fuel smoke during pregnancy on birth outcomes. A pre-intern doctor visited each household and recruited the children. At recruitment, parents or guardians of the child were informed of the objectives of the study and the procedures involved; written consent was obtained from the parents or guardians prior to recruitment. Children who fulfilled inclusion and exclusion criteria were recruited into the study.

An interviewer administered questionnaire, a symptom diary, and a time activity pattern data sheet were specifically developed for data collection. The interviewer administered questionnaire was administered to the mother on recruitment of the child. The parents were explained on how to maintain the symptom diary. The symptom diary was used to obtain information on whether children had any respiratory symptoms on a given day. The diary was kept with the parents; seven (07) symptoms including fever, sore throat, rhinitis, rhinoconjunctivitis, sneezing, cough, and wheezing were assessed. Parents were requested to mark any symptom that the child had on a particular day. Households were visited on a random basis to determine if the symptom diary was properly filled. Data extracted from the symptom diary were collected from households every month by research assistants during home visits.

The respiratory health status of children was obtained by a questionnaire adapted from the translated and validated ISAAC questionnaire [15] used in Sri Lanka and the American Thoracic Society questionnaire [16]. This was translated from English to Sinhala and re-translated back to English by an independent person; the two English versions were compared and necessary adjustments were made.

Information on congenital defects or syndromic conditions, having siblings, growth deficiencies, attending a pre-school or day care center, overcrowding, cigarette smoking inside the house, presence of other industries causing air pollution near the house, parental education, parental occupation and monthly income were also obtained.

The questionnaire was pretested on 10 mothers in the area. Shortcomings in the questionnaires were corrected and revised accordingly.

Children who were living in households where biomass fuel or kerosene oil was used as the principal type of cooking fuel, were classified as the high exposure group. Children living in households where LPG or electricity was used as the principal type of cooking fuel, were classified as the low exposure group.

An upper respiratory tract infection (URTI) was defined as having two of the following symptoms including sore throat, cough, runny nose, fever > 38 °C for one or more days and a physician diagnosis of an upper respiratory tract infection [17]. Lower respiratory tract infections (LRTI) were defined as having fever > 38 °C and cough with purulent sputum and rales in the lungs [12]. Infection induced asthma was defined as having shortness of breath or dry cough or wheezing for 2 or more days after fever had subsided. Exacerbation of asthma was defined as dry cough or wheezing without fever for 2 or more days. Rhinitis was defined as sneezing or having a runny nose without fever for 2 or more days.

Air quality measurements were recorded in a subsample of households which were selected based on recruitment to the parent study. Forty percent of households of pregnant females were selected for air quality monitoring. PM_2.5_ levels and the CO concentrations were measured using two real time monitors: PM_2.5_ levels were measured using TSI’s new 8530 DustTrak II aerosol monitor and carbon dioxide (CO_2_) and carbon monoxide (CO) concentrations were measured using TSI’s 7575 Q-Trak™ indoor air quality monitor. Air quality measurements were recorded in 125 households. Measurements were done for two consecutive hours with minute-to-minute recording during preparation of lunch. In Sri Lanka, the main meal prepared in the house is lunch and the duration a stove is lit for this purpose is between 1 and 2 h. Therefore 2 h consecutive measurements during preparation of lunch were obtained for assessment of household air quality. Standard guidelines were followed while mounting the probes to minimize errors in measurements. Before installing the air quality measuring monitors, the data collector inspected the vicinity and the places of installation. If it was not possible to set up the instruments according to manufacturer’s specifications given in the guidelines, necessary physical changes were made. The receivers/inlet of the monitors were kept at 145 cm above the floor and 100 cm from the cook stove with not more than a 10 cm difference from specified standards. Monitors were placed with the receivers/ inlet at least 150 cm away from windows and doors (openings). Guidelines were adhered to using a measuring tape for measuring distances. PM_2.5_ levels were corrected against a gravimetric measurement using a correction factor [18].

### Data analysis

Data were entered into EPIDATA data bases (separately for each source of data) and analyzed using SPSS version 16 software and Winpepi software. Categorical data were analyzed using chi square tests, odds ratios and their 95% confidence intervals.

In the follow up study, the incidence of the respiratory diseases between different exposure categories was compared. Incidence rates were calculated as the number of episodes per thousand child-months of observation. Rate ratios and their 95% confidence intervals were calculated using Winpepi software.

Measurements of PM_2.5_, CO and CO_2_ levels were compared between the two exposure groups using the independent sample t-test. Poisson regression analysis was used to identify risk factors of infection induced asthma. All variables associated with infection induced asthma and exposure status on bivariate analysis were included in the final model.

## Results

The study population comprised 262 children at baseline of whom 54% were males (Table 1). The majority were Sinhalese comprising 93% of the population; 5% were Tamil and the rest were of Burgher and Moor origin. Sixty percent of children were residing in houses using firewood or kerosene oil as the principal fuel for cooking (high exposure group). Parental education levels (*p* = 0.02 for paternal education and *p* = 0.01 for maternal education) and family income (*p* = 0.017) were significantly different in the two exposure groups (high and low) at baseline.

**Table 1:** Socio demographic characteristics of the study population at baseline

Characteristic	High exposure group^a^	Low exposure group^b^	*P*-value
Sex			
Male	85 (54.1)	57 (54.3)	0.541
Female	72 (45.9)	48 (45.7)	
Age group			
Up to 1 year	5 (3.3)	4 (3.9)	0.365
1.01-2 years	26 (16.9)	28 (27.5)	
2.01–3 years	53 (34.5)	29 (28.4)	
3.01–4 years	37 (24.2)	22 (21.6)	
4.01–5 years	32 (21.1)	19 (18.6)	
Ethnicity			
Sinhala	149 (95.5)	96 (90.5)	0.179
Other	7 (4.5)	10 (9.5)	
Father’s education			
Up to O/L^*^	112 (72.2)	63 (60.0)	0.027
Above O/L	43 (27.8)	42 (40.0)	
Mother’s education			
Up to O/L	106 (68.4)	56 (53.3)	0.010
Above O/L	49 (31.6)	49 (46.7)	
Family income			
Up to SLR 20,000	42 (27.1)	16 (15.2)	0.017
More than SLR 20,000	113 (72.9)	89 (84.8)	
Mother’s employment			
Yes	2 (1.3)	4 (4.0)	0.175
No	147 (98.7)	95 (96.0)	
Presence of industries causing air pollution in vicinity of house			
Yes	32 (21.3)	18 (17.1)	0.253
No	118 (78.7)	87 (82.9)	
Child’s room			
Directly opens to kitchen	27 (17.8)	17 (17.7)	0.567
Not directly opened to kitchen	125 (82.2)	79 (82.3)	
Having a chimney			
Yes	79 (58.9)	36 (41.8)	0.010
No	55 (41.1)	50 (58.2)	
Place of cooking			
Inside the living area	7 (4.8)	4 (4.2)	0.528
Outside the living area	136 (95.2)	92 (95.8)	
Cooking frequency			
Up to 10 times per week	110 (75.8)	68 (71.5)	0.276
Equal to or greater than 10 times per week	35 (24.2)	27 (28.5)	
Duration of cooking			
Up to 2.5 h per day	70 (46.7)	67 (67.0)	0.001
Greater than 2.5 h per day	80 (53.3)	33 (33.0)	
Ventilation			
Window area > 1/7 of floor area	122 (90.4)	87 (95.6)	0.141
Window area < 1/7 of floor area	13 (9.6)	4 (4.4)	
Either one of the parents having asthma			
Yes	77 (52.0)	43 (44.7)	0.165
No	71 (48.0)	53 (55.3)	
Having pets			
Yes	48 (32.0)	36 (36.7)	0.263
No	102 (68.0)	62 (63.3)	
Having a sibling			
Yes	94 (60.6)	48 (45.7)	0.012
No	61 (39.4)	57 (54.3)	
Attending a Preschool			
Yes	60 (39.7)	41 (39.8)	0.509
No	91 (60.3)	62 (60.2)	
Having a smoker at home			
Yes	39 (25.5)	24 (23.1)	0.386
No	114 (74.5)	80 (76.9)	

^*^O/L Ordinary level exam, *SLR* refers to Sri Lankan Rupees (1 USD ~ 130 SLR at time of study)

^a^Children exposed to biomass and kerosene as the principal cooking fuel

^b^Children exposed to LPG and electricity as thee principal cooking fuel

Asthma in parents, attending a pre-school, having a pet or having a smoker at home were distributed evenly in the two exposure groups; having a sibling was significantly more common in the high exposure group (*p* = 0.01).

Of the 262 children who were initially recruited, 20 were lost during follow up. Parents of nine children withdrew consent just after recruitment; parents of one child withdrew consent during follow up. Ten children changed their residence and were lost to follow-up.

The prevalence of respiratory symptoms was assessed at recruitment. Exposure status was not associated with ever wheezing, past history of physician diagnosed asthma, nocturnal dry cough, exercise induced asthma, sneezing, rhinitis, cough with cold and phlegm, and cold (data not shown).

Children living in families having a monthly income of SLR 20,000 (I USD ≈ SLR 135 during the time of the study) or less were almost 3 times more likely to have a past history of sneezing (OR = 2.84; 95% CI = 1.33–6.05) and 0.5 times less likely to have a past history of cough with cold (OR = 0.5; 95% CI = 0.24–0.95) than children from families with a monthly family income greater than SLR 20,000. Lower maternal education was significantly associated with having a past history of phlegm with cold (OR = 1.8; 95% CI = 1.09–3.05) as compared to children of mothers who were educated more than Ordinary level (O/L). Having a sibling increased the likelihood of a child having a past history of having phlegm with cold almost two-fold (OR = 1.96; 95% CI = 1.19–3.24) as compared to a child without siblings. Children living in households in which cooking is done within 2.5 h had a significantly lower likelihood of having a past history of physician diagnosed asthma (OR = 0.5; 95% CI = 0.24–0.99) as compared to children in households where the duration of cooking is more than 2.5 h a day. Children attending a pre-school or daycare center were more than twice as likely to have a past history of physician diagnosed asthma, nocturnal dry cough and rhinitis (OR = 2.12, 2.81, 2.67, respectively) as compared to children not attending a pre-school or daycare center. Having a family history of asthma significantly increased the likelihood of children having wheezing, asthma, nocturnal dry cough, exercise induced asthma, sneezing and rhinitis in the past (Table 2).

**Table 2:** Respiratory symptoms and socio demographic characteristics of the study population (Unadjusted results)

Characteristics	Symptom																	
	Ever wheezing			Asthma			Sneezing			Rhinitis			Cough & cold			Phlegm & cold		
	Yes n (%)	No n (%)	OR^a^ (95% CI)	Yes n (%)	No n (%)	OR^a^ (95% CI)	Yes n (%)	No n (%)	OR^a^ (95% CI)	Yes n (%)	No n (%)	OR^a^ (95% CI)	Yes n (%)	No n (%)	OR^a^ (95% CI)	Yes n (%)	No n (%)	OR^a^ (95% CI)
Family income																		
Up to SLR 20,000	21 (22.8)	35 (21.0)	1.107 (0.60–2.04)	5 (11.9)	51 (23.7)	0.43 (0.16–1.16)	14 (40.0)	42 (19.0)	2.84 (1.34–6.05)	8 (38.1)	48 (20.3)	2.41 (0.95–6.15)	40 (19.1)	16 (33.3)	0.47 (0.24–0.95)	34 (22.7)	22 (20.4)	1.15 (0.63–2.10)
More than SLR 20,000	71 (77.1)	131 (78.9)		37 (88.1)	164 (76.3)		21 (60.0)	179 (81.0)		13 (61.9)	188 (79.7)		169 (80.9)	32 (66.7)		116 (77.3)	86 (79.6)	
Mother’s education																		
Up to O/L	61 (66.3)	99 (59.6)	1.33 (0.78–2.26)	25 (59.5)	135 (62.8)	0.87 (0.44–1.71)	22 (14.5)	138 (62.4)	1.02 (0.49–2.13)	12 (57.1)	146 (61.9)	0.79 (0.32–1.96)	130 (62.2)	29 (60.4)	1.08 (0.57–2.05)	102 (68.0)	58 (53.7)	1.83 (1.09–3.05)
Above O/L	31 (33.6)	67 (40.4)		17 (40.5)	80 (37.2)		130 (85.5)	83 (37.6)		9 (42.9)	90 (38.1)		79 (37.8)	19 (39.6)		48 (32.0)	50 (46.3)	
Either one of the parents having asthma																		
Yes	50 (60.2)	70 (43.5)	1.97 (1.15–3.38)	24 (66.7)	96 (46.4)	2.31 (1.1–4.87)	22 (68.7)	98 (46.7)	2.51 (1.14–5.57)	16 (88.9)	103 (45.8)	9.48 (2.13–42.18)	102 (51.0)	18 (40.9)	1.50 (0.78–2.91)	78 (53.4)	42 (42.9)	1.53 (0.53–2.38)
No	33 (39.8)	91 (56.5)		12 (33.3)	111 (53.6)		10 (31.3)	112 (53.3)		02 (11.1)	122 (54.2)		98 (49.0)	26 (59.1)		68 (46.6)	56 (57.1)	
Total cooking hours																		
Less than 2.5 h	45 (51.7)	90 (55.9)	0.85 (0.50–1.43)	15 (39.5)	119 (56.9)	0.49 (0.24–0.99)	14 (42.4)	120 (56.1)	0.58 (0.28–1.21)	10 (50.0)	125 (55.1)	0.82 (0.33–2.04)	110 (55.0)	25 (53.2)	1.08 (0.57–2.03)	77 (54.2)	58 (54.7)	0.98 (0.59–1.63)
2.5 h or more	42 (48.3)	71 (44.1)		23 (60.5)	90 (43.1)		19 (57.6)	94 (43.9)		10 (50.0)	102 (44.9)		90 (45.0)	22 (46.8)		65 (45.8)	48 (45.3)	
Attending a preschool																		
Yes	42 (44.7)	61 (36.7)	1.39 (0.83–2.33)	23 (54.8)	79 (36.4)	2.12 (1.09–4.12)	19 (54.3)	83 (37.2)	2.00 (0.98–4.11)	13 (61.9)	90 (37.8)	2.67 (1.06–6.70)	80 (37.9)	22 (45.8)	0.72 (0.38–1.36)	55 (36.4)	48 (44.0)	0.73 (0.44–1.20)
No	52 (55.3)	105 (63.3)		19 (45.2)	138 (63.6)		16 (45.7)	140 (63.8)		8 (29.1)	148 (62.2)		131 (62.1)	26 (54.2)		96 (63.6)	61 (56.0)	
Having a sibling																		
Yes	54 (57.4)	88 (53.0)	1.19 (0.71–1.99)	24 (57.1)	117 (53.9)	1.14 (0.59–2.22)	21 (60.0)	120 (53.8)	1.29 (0.62–2.66)	12 (57.1)	130 (54.6)	1.11 (0.45–2.73)	117 (55.5)	24 (50.0)	1.24 (0.67–2.33)	93 (61.6)	49 (45.0)	1.96 (1.19–3.24)
No	40 (42.6)	78 (47.0)		18 (42.9)	100 (46.1)		14 (40.0)	103 (46.2)		9 (42.9)	108 (45.4)		94 (44.5)	24 (50.0)		58 (38.4)	60 (55.0)	

^a^ Unadjusted odds ratio

On bivariate analysis, the incidence of respiratory tract infections and infection induced asthma were significantly higher among children in the high exposure group as compared to children in the low exposure group (RR = 1.35 and 2.03, respectively) (Table 3). The incidence of asthma attacks, rhinitis and rhinoconjunctivitis exacerbations were not associated with exposure status.

**Table 3:** Respiratory diseases and exposure group

Respiratory diseases	High exposure group^d^	Low exposure group^e^	RR (95% CI)
URTI^a^			
Number of episode	91	61	1.03 (0.74–1.45)
Total child months	1768	1218	
Incidence Rate (Number of episodes / 1000 months of observation)	51.5	50.1	
LRTI^b^			
Number of episodes	166	70	1.63 (1.23–2.19)
Total child months	1768	1218	
Incidence Rate (Number of episodes / 1000 months of observation)	93.9	57.5	
RTI^c^			
Number of episodes	257	131	1.35 (1.09–1.68)
Total child months	1768	1218	
Incidence Rate (Number of episodes / 1000 months of observation)	145.4	107.6	
Asthma			
Number of episodes	378	264	0.99 (0.84–1.16)
Total child months	1768	1218	
Incidence Rate (Number of episodes / 1000 months of observation)	213.8	216.7	
Infection induced asthma			
Number of episodes	124	42	2.03 (1.42–2.96)
Total child months	1768	1218	
Incidence Rate (Number of episodes / 1000 months of observation)	70.1	34.5	
Rhinitis			
Number of episodes	249	181	0.95 (0.78–1.16)
Total child months	1763	1217	
Incidence Rate (Number of episodes / 1000 months of observation)	141.2	148.7	

^a^ refers to upper respiratory tract infections, ^b^ refers to lower respiratory tract infection and ^c^refers to respiratory tract infections including both URTI and LRTI, ^d^ Children exposed to biomass fuel and kerosene oil as the principal type of cooking fuel ^e^ Children exposed to LPG and electricity as the principal type of cooking fuel

The incidence of asthma among males was significantly higher than in females (RR = 1.17; 95% CI 1.01–1.37). Having an industry releasing air pollutants near the house and cooking inside the living area were significant risk factors of rhinitis while spending less time on cooking was a protective factor (RR = 1.39, 2.67, 0.81, respectively). Having a sibling, attending pre-school, having a pet, and monthly family income were not associated with the incidence of respiratory diseases/conditions (Table 4).

**Table 4:** Respiratory diseases and associated factors

Characteristics	Incidence of diseases							
	LRTI		Infection induced asthma		Asthma		Rhinitis	
	Incidence rate * 1000 (number of episodes/ months of observation)	Rate Ratio (95% CI)	Incidence rate * 1000 (number of episodes/ months of observation)	Rate Ratio (95% CI)	Incidence rate * 1000 (number of episodes/ months of observation)	Rate Ratio (95% CI)	Incidence rate * 1000 (number of episodes/ months of observation)	Rate Ratio (95% CI)
Cooking								
Inside living area	79.5 (141/1773)	0.94 (0.72–1.25)	51.3 (91/1773)	0.76 (0.55–1.06)	213.9 (379/1772)	0.98 (0.83–1.16)	232.9 (413/1773)	2.67 (2.11–3.40)
Outside living area	84.3 (84/997)		67.2 (67/997)		218.5 (217/993)		87.3 (87/997)	
Cooking hours								
Up to 2.5 h /day	72.8 (114/1565)	0.83 (0.63–1.08)	50.5 (79/1565)	0.79 (0.57–1.08)	219.0 (342/1562)	1.06 (0.90–1.25)	130.4 (204/1564)	0.81 (0.67–0.99)
Greater than 2.5 h /day	88.0 (115/1307)		64.3 (84/1307)		206.6 (270/1307)		160.5 (209/1302)	
Having an industry near home								
Yes	77.7 (102/1312)	0.97 (0.74–1.27)	51.8 (68/1312)	0.89 (0.64–1.23)	210.1 (275/1309)	0.95 (0.81–1.11)	174.8 (229/1310)	1.39 (1.15–1.69)
No	79.9 (129/1614)		58.2 (94/1614)		222.1 (358/1612)		125.5 (202/1610)	
Sex								
Male	77.6 (124/1598)	0.96 (0.74–1.25)	49.4 (79/1598)	0.79 (0.57–1.08)	230.9 (369/1598)	1.17 (1.00–1.37)	140.6 (224/1593)	0.93 (0.77–1.13)
Female	80.7 (112/1388)		62.7 (87/1388)		197.4 (273/1383)		151.4 (210/1387)	
Having a sibling								
Yes	84.0 (139/1655)	1.13 (0.87–1.49)	64.0 (106/1655)	1.38 (0.99–1.93)	223.2 (369/1653)	1.09 (0.93–1.28)	146.5 (242/1652)	0.99 (0.82–1.21)
No	74.1 (96/1295)		46.3 (60/1295)		204.3 (264/1292)		147.1 (190/1292)	
Attending to preschool								
Yes	74.8 (88/1177)	0.90 (0.68–1.18)	59.5 (70/1177)	1.10 (0.80–1.51)	213.3 (251/1177)	0.990 (0.84–1.16)	144.8 (170/1174)	0.98 (0.81–1.20)
No	82.9 (147/1773)		54.1 (96/1773)		216.0 (383/1773)		147.5 (261/1770)	
Having a pet								
Yes	76.1 (74/972)	0.93 (0.69–1.23)	53.5 (52/972)	0.89 (0.63–1.25)	225.3 (219/972)	1.08 (0.91–1.28)	135.8 (132/972)	1.03 (0.83–1.27)
No	82.0 (153/1866)		60.0 (112/1866)		208.0 (387/1861)		132.3 (246/1860)	
Family income								
Up to SLR 20,000	79.3 (55/694)	1.00 (0.73–1.36)	56.2 (39/694)	1.01 (0.69–1.46)	219.7 (152/692)	1.03 (0.85–1.23)	160.4 (111/692)	1.14 (0.91–1.41)
Greater than SLR 20,000	79.0 (181/2292)		55.4 (127/2292)		214.1 (490/2289)		141.2 (323/2288)	

Houses which used biomass fuel for cooking had significantly higher concentrations of CO (*p* = 0.002) and PM_2.5_ (*p* < 0.001) as compared to houses using LPG and electricity (Table 5). There was no difference in CO_2_ concentrations between houses using biomass fuel and LPG/electricity for cooking.

**Table 5:** Air quality measurements in selected houses

Exposure	Number of households	Mean	SD	*P*-value
CO				
High exposure^a^	64	2.77 ppm	2.63 ppm	0.002
Low exposure^b^	51	1.44 ppm	1.60 ppm	
PM_2.5_				
High exposure^a^	66	0.62 mg/m^3^	0.99 mg/m^3^	< 0.001
Low exposure^b^	52	0.19 mg/m^3^	0.27 mg/m^3^	
CO_2_				
High exposure^a^	65	558.6 ppm	120.1 ppm	0.671
Low exposure^b^	56	549.8 ppm	111.2 ppm	

^a^ Children exposed to biomass fuel and kerosene oil as the principal type of cooking fuel

^b^ Children exposed to LPG and electricity as the principal type of cooking fuel

PM_2.5_ and carbon dioxide in the ambient air was positively correlated with incidence of lower respiratory tract infections (*p* = < 0.001and *p* = 0.028, respectively). Infection induced asthma was positively correlated with PM_2.5_ levels (*p* < 0.001) (Table 6).

**Table 6:** Correlation coefficients between air quality measurements and respiratory illnesses

Respiratory illness (disease episodes per year)	Air Pollutant (mean level of measured air pollutant during cooking)					
	CO (*n* = 105)		CO_2_ (*n* = 111)		PM_2.5_ (*n* = 113)	
	Pearson Correlation Coefficient	*p*-value	Pearson Correlation	*p*-value	Pearson Correlation	*p*-value
RTI	0.092	0.352	0.89	0.355	0.256	0.006
LRTI	0.168	0.092	0.211	0.028	0.327	< 0.001
Asthma	−0.026	0.799	0.002	0.980	−0.077	0.429
Infection induced Asthma	0.107	0.278	0.113	0.236	0.327	< 0.001

*RTI* Respiratory tract infection, *LRTI* Lower respiratory tract infection, *CO* Carbon monoxide, *CO*_*2*_ Carbon dioxide, *PM*_*2.5*_ Particulate matter 2.5 μm

Using a Poisson regression analysis, living in a house using biomass fuel or kerosene for cooking (high exposure) and having a mother educated below O/L were significant predictors of infection induced asthma after controlling for father’s education, family income, duration of cooking, having a sibling and the kitchen having a chimney (Table 7). Children resident in high exposure houses were 1.7 times more likely to experience an episode of infection induced asthma as compared to children living in low exposure houses; children whose mothers were less educated (up to O/L) were 2.1 times more likely to experience an episode of infection induced asthma as compared to children whose mothers were more educated (beyond O/L) after controlling for other variables.

**Table 7:** Summary of Poisson regression analysis using infection induced asthma as the dependent variable

Variable	Regression Coefficient	Std. Error of regression coefficient	Adjusted Relative Risk (95% CI)
Intercept	−0.098	0.5282	
High exposure^a^	0.572	0.2510	1.772 (1.098–2.949)
Father’s education (up to O/L)^b^	−0.494	0.2677	0.610 (0.362–1.037)
Mother’s education (up to O/L)^c^	0.778	0.2774	2.177 (1.276–3.803)
Family income (< SLR 20,000)^d^	−0.196	0.2701	0.822 (0.475–1.374)
Duration of cooking (< 2.5 h)^e^	−0.343	0.2277	0.710 (0.452–1.106)
Having a chimney^f^	−0.294	0.2212	0.745 (0.482–1.150)
Having a sibling^g^	−0.202	0.2422	1.224 (0.764–1.982)

^a^Reference group is low exposure group using LPG and electricity for cooking

^b^Reference group is father’s education above ordinary level (O/L)

^c^Reference group is mother’s education above ordinary level (O/L)

^d^Reference group is having income of Sri Lanka Rupees (SLR) 20,000 or more

^e^Reference group is the households where they spent 2.5 h or more for cooking

^f^Reference group is households without a chimney

^g^Reference group is the children without a sibling

## Discussion

This study was carried out in a semi urban mixed population in Sri Lanka where biomass fuel and kerosene use as the main cooking fuel is still high. Our results show that exposure to household air pollution due to biomass fuel or kerosene oil usage significantly increases the risk of self reported lower respiratory tract infections and infection induced asthma in children under 5. In addition, low maternal education was a significant predictor of infection induced asthma after controlling for other potential confounders.

It has been shown that the incidence of infection induced asthma and respiratory tract infections is higher in children of households using biomass or kerosene for cooking as compared to children of households using LPG or electricity after controlling for other variables. WHO has estimated the incidence of lower respiratory tract infections as 0.29/child/year in developing countries while it is 0.05/child/year in developed countries [19]. In this study, the incidence of lower respiratory tract infections was 0.95/child/year which is much higher than the WHO estimate especially in children of households using biomass or kerosene for cooking. As expected, the incidence of lower respiratory tract infections was less in children of households using LPG or electricity for cooking; the overall incidence of LRTI was 0.69/child/year.

Our results are in agreement with published literature [7, 8] and the mounting evidence on the health hazards of household air pollution especially on respiratory health [20]. While CO levels in households using biomass fuel was almost twice as much as in households using LPG and electricity, PM_2.5_ levels were 3.5 times higher.

Socio-economic characteristics of households using biomass or kerosene oil as the main cooking fuel were significantly different to households using LP gas or electricity. As expected, households in which parents were more educated and had a higher monthly income were more likely to use LPG or electricity for cooking. With use of cleaner fuels (LPG and electricity), the duration of cooking is also significantly reduced further mitigating the exposure to household air pollutants. Cooking patterns in the two exposure groups were similar and most mothers were housewives. Children of households using biomass or kerosene were more likely to have a sibling as compared to children of households using LPG or electricity.

Maternal education has been shown to be a predictor of childhood morbidity and mortality [21] including respiratory tract infection induced asthma [20]. In our study, a child whose mother was educated less than O/L increased the likelihood of the child acquiring an infection induced asthma episode by two-fold as compared to a child whose mother was educated beyond O/L. The independent effect of maternal education was seen even after controlling for other variables including exposure status probably reflecting the wider impact of maternal education on health of children, in general.

Asthma, a condition known to have a genetic predisposition, is significantly higher among children of asthmatic parents [22, 23]. In this study, having a history of physician diagnosed asthma, rhinitis and sneezing were significantly higher among offspring of asthmatic parents. However, none of these clinical entities were associated with exposure status. A systematic review revealed that there is no significant association between asthma and household air pollution [11].

Not having a sibling was a protective factor for respiratory tract infections. It has been reported previously that children with siblings are almost twice as likely to experience respiratory symptoms of phlegm and cold as compared to children without siblings [24]. Most children in our sample had elder siblings who were attending a school or pre-school; as expected, these siblings tended to bring infections from schools, probably of viral origin, and pass them on to other siblings.

In our study, asthma and rhinitis were significantly higher among children attending pre-schools or daycare centers as compared to children staying at home. Rhinitis is an inflammatory disorder of the nasal mucosa characterized by nasal congestion, rhinorrhea and itching, often accompanied by sneezing and conjunctival irritation due to irritation of the respiratory mucosa by a particular pollutant [25]. Children attending pre-schools and daycare centers get exposed to different environments and are exposed to new allergens like dust and pollen which may be the trigger for episodes of asthma and rhinitis.

Physician diagnosed asthma was commoner among children from households that cooked meals for longer periods of time. While this is probably confounded by the cooking fuel used, the shorter exposure to possibly fewer pollutants may partly explain the difference in the prevalence of physician diagnosed asthma in the two groups.

During the follow up period of 12 months, respiratory symptoms in children were recorded on a daily basis. The incidence of respiratory tract infections and infection induced asthma were significantly higher among children of households using biomass or kerosene. Cooking inside the living area, longer cooking time and having an industry emitting pollutants near a child’s house, all of which are known to increase air pollutant levels, significantly increased the occurrence of rhinitis episodes.

In our study, asthma was more common among male children as reported previously [26]. Having a sibling, having a pet, monthly income or going to pre-school were not associated with incident episodes of respiratory tract infections. The duration of follow up in this study may have been inadequate to elicit a relationship between incidence of respiratory tract infections and these variables.

Air quality measurements done in a subsample of households showed significantly higher levels of PM_2.5_ and CO in households using biomass fuel as compared to households using LPG or electricity. Air quality monitoring was limited to a select number of houses due to the difficulty in carrying out the procedure. There is unequivocal evidence that household air pollution caused by incomplete combustion of biomass fuels is a major health hazard [27, 28]. Our findings confirm that even in the Sri Lankan setting the levels of pollutants in households using biomass fuel as the main cooking fuel is much higher than in households using cleaner fuels. A limitation of our study was not considering the use of secondary fuel. We did not consider this, as when air quality monitoring was done, almost all the houses were in accordance with the initial categorization based on the baseline questionnaire data.

We did not observe a significant difference in carbon dioxide levels in the households of the two exposure groups although it has been reported that carbon dioxide emissions are higher in houses using biomass fuel than in houses using LPG. Carbon dioxide emissions of a fuel during the combustion process depend on the carbon content of the type of fuel used.

PM_2.5_ levels were significantly and positively correlated with the number of incident respiratory tract infections and episodes of infection induced asthma as recorded in the literature. Inhalation of fine particles of small size causes more damage as they penetrate deep into the lungs and may enter the blood stream [29].

Although the major strength of this study is being a longitudinal one in which children were monitored on a regular basis over a 12-month period and incidences of symptom/ disease episodes were recorded, there are a few limitations, some of which have already been highlighted, that need to be considered in the overall interpretation of the findings. We used self reported data on respiratory symptoms without confirmation by a clinician which was the only way out given the nature of the symptoms and health care seeking behavior for such symptoms among the general public; as the children were monitored every month by the research team, we do not expect this to have much of an impact on our estimates, most of which are similar to findings previously reported.

We were able to monitor air quality only in a subsample of households, over a two-hour period during the preparation of the main lunch meal, which revealed higher concentrations of pollutants in houses using biomass and kerosene for cooking. This two-hour measurement during the preparation of the lunch meal may not reflect the actual exposure to indoor air pollutants resulting from cooking over a 24-h period; it is likely that households cook more than once a day and inhabitants are exposed to higher concentrations of air pollutants over a 24-h period that includes exposure to residual pollutants after cooking. As practices in Sri Lankan households are similar, we believe that our findings are representative of all households.

We classified children of households using biomass and kerosene as the “high exposure” group and children of households using LPG and electricity as the “low exposure” group, based on information obtained at baseline. It is possible that some households may have switched their energy source during the study. However, when air quality measurements were made in the subsample of households during the study, none of the households classified at baseline had changed their energy source for cooking; hence we surmise that our original classification of households is acceptable and likely to not have changed as there was no significant economic implications in terms of prices of different energy sources or the socio-economic status of families during the 12-month study period.

## Conclusion and recommendations

CO and PM_2.5_ concentrations were significantly higher in households using biomass fuel for cooking. There was a 1.6 times higher risk of LRTI and two times higher risk of infection induced asthma among children of households using biomass fuel and kerosene for cooking as compared to children of households using LPG or electricity, after adjusting for confounders. Use of cleaner fuels for cooking is recommended: if there are economic constraints, it is recommended that children are kept away from stoves, preferably outside the kitchen, while the stoves are lit.

## Data Availability

The datasets used and analyzed during the current study are available from the corresponding author on request. As we have not completed the analysis, we cannot present the data within the manuscript.

## Abbreviations

ARI: Acute respiratory tract infections
CI: Confidence interval
CO: Carbon monoxide
ISAAC: International Study of Asthma and Allergies in Childhood
ITREOH: International Training and Research in Environmental and Occupational Health
LPG: Liquefied Petroleum Gas
LRTI: Lower respiratory tract infections
MOH: Medical Officer of Health
PM_2.5_: Particulate matter 2.5
RR: Rate ratio
RTI: Respiratory tract infections
SLR: Sri Lankan Rupees
URTI: Upper respiratory tract infections
WHO: World Health Organization
